# Supplementary material for: Accurate localization microscopy by intrinsic aberration calibration
Source: Nat Commun. 2021 Jun 24;12:3925. doi: 10.1038/s41467-021-23419-y (PMC8225824; doi:10.1038/s41467-021-23419-y)
Supplement: Supplementary file 1 — Supplementary Information [file 41467_2021_23419_MOESM1_ESM.pdf]

Supplementary Information

Accurate Localization Microscopy by Intrinsic Aberration Calibration

Craig R. Copeland,[1] Craig D. McGray,[2] B. Robert Ilic[1,3], Jon Geist,[2] and Samuel M. Stavis[1,*]

[1]Microsystems and Nanotechnology Division, National Institute of Standards and Technology, Gaithersburg, MD, USA
[2]Quantum Measurement Division, National Institute of Standards and Technology, Gaithersburg, MD, USA
[3]CNST NanoFab, National Institute of Standards and Technology, Gaithersburg, MD, USA
*Address correspondence to samuel.stavis@nist.gov.

INDEX

Supplementary Table 1. Optical microscopy and interferometry methods to measure micromechanical motion and surface topography
Supplementary Figure 1. Particle images and Gaussian fits
Supplementary Table 2. Model functions for localization
Supplementary Figure 2. Localization scheme
Supplementary Note 1. Apparent lateral motion
Supplementary Figure 3. Field curvature
Supplementary Figure 4. Plot showing the dependence of the parameter ρ_w on axial position.
Supplementary Figure 5. Field dependence of local values of uncertainty from axial localization for ρ and ρ_w
Supplementary Figure 6. Field dependence of local values of uncertainty for Δ'_x and Δ'_y
Supplementary Figure 7. Lateral dependence of apparent lateral motion
Supplementary Figure 8. Aberrations and sample tilt can limit axial range
Supplementary Figure 9. Variation of aberration effects at micrometer scales
Supplementary Figure 10. Zernike polynomial coefficients
Supplementary Figure 11. Lateral widefield calibration errors for Zernike polynomials
Supplementary Figure 12. Axial widefield calibration errors for Zernike polynomials
Supplementary Figure 13. Lateral widefield calibration errors for natural neighbor interpolation
Supplementary Figure 14. Axial widefield calibration errors for natural neighbor interpolation
Supplementary Note 2. Rigid transformation model
Supplementary Figure 15. Residuals of rigid transformations and uncertainties of single particles
Supplementary Figure 16. Apparent lateral motion
Supplementary Table 3. Comparison of transformation residuals and localization uncertainty
Supplementary Figure 17. Uncertainty evaluation for rigid transformations
Supplementary Table 4. Transformation residuals from a representative simulation
Supplementary Note 3. Field corrections
Supplementary Figure 18. Flexure of the load gear
Supplementary Figure 19. Rotational play produces a reciprocating vertical shift
Supplementary Figure 20. Rotational play out of the plane
Supplementary Figure 21. Emission spectra
Figure 22. Balance of speed and precision

Supplementary Table 1. Optical microscopy and interferometry methods to measure micromechanical motion and surface topography

Measurement method	Degrees of freedom	Translational resolution		Rotational resolution		Translational range		Rotational range		Surface topography	Contrast mechanism	Accessibility $(\text{complexity} \times \text{cost})^{-1}$
		x, y **(nm)**	***z*** **(nm)**	γ^a **(µrad)**	α, β **(mrad)**	***x, y*** **(µm)**	***z*** **(µm)**	γ^a **(rad)**	α, β **(rad)**			
**Localization Microscopy**	*x, y, z, α, β, γ*	< 1 to 10	10 to 100	< 1	1 to 100	> 250	10	2π	2π[d]	*x, y, z*	Particle, edge, hole	High
**Image correlation microscopy**	*x, y*[b]	10 to 1,000	—	—[b]	—[b]	> 250	—	—[b]	—[b]	*x, y*	Particle, edge, hole	High
**Digital holographic microscopy**	*x, y, z*	1000	< 1	—[b]	—[b]	> 250	10	—[b]	—[b]	*x, y, z*	Surface	Low
**White-light interferometric microscopy**	*x, y, z*	1000	< 1	—[b]	—[b]	> 250	>100[e]	—[b]	—[b]	*x, y, z*	Surface	Low
**Laser doppler vibrometry**	*x, y, z*	< 1	< 1	—	—	10[c]	10[c]	—	—	—	Reflective surface	Low

[a] For $\beta = 0$ such that the mixed coordinate system of this study equals the camera coordinate system
[b] It is possible to measure γ but we are unaware of any study that has reported such analysis
[c] Point measurements require lateral scanning across the surface of a planar device, and insufficient surface roughness may degrade or prevent measurement
[d] The measurement range for z may limit the range for rotations out of plane, depending on the extent of the emitter constellation
[e] Requires axial scanning

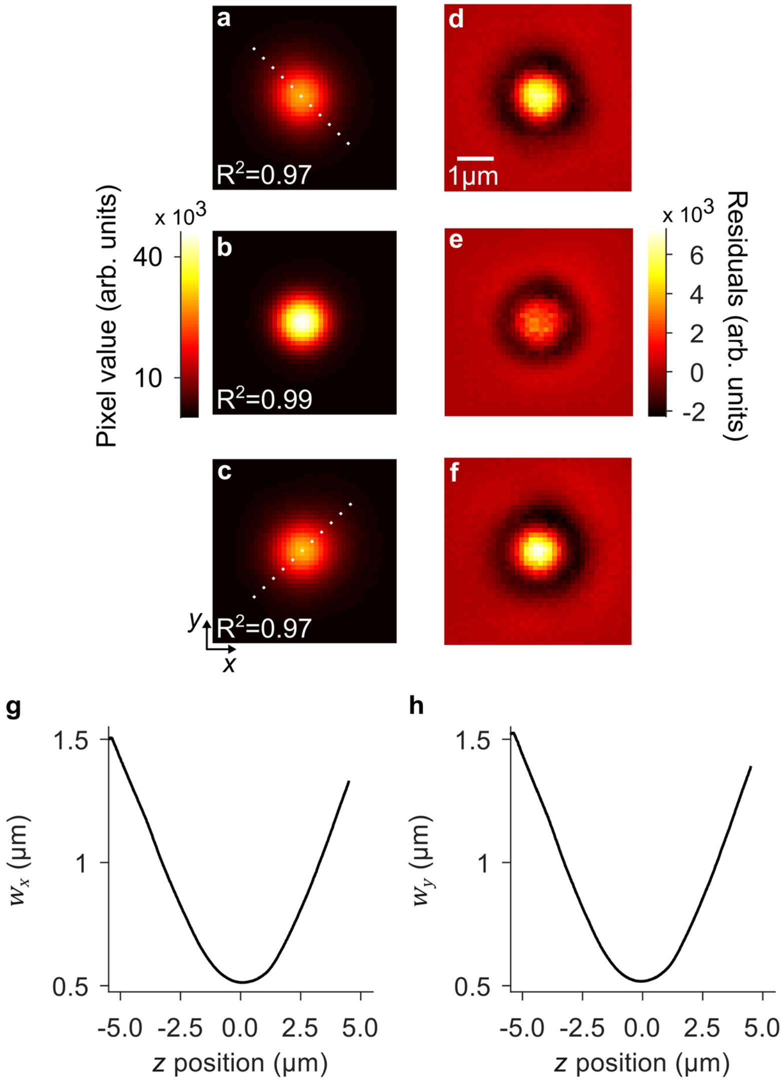

Supplementary Figure 1. Particle images and Gaussian fits. Details of the model are in Supplementary Table 2. (**a-c**) Optical micrographs (false color) showing images of a fluorescent particle at z positions of (a) 2 µm above best focus, (b) near best focus, and (c) 2 µm below best focus. Two effects of intrinsic aberrations are apparent – symmetry variation due to astigmatism and intensity variation due to defocus. Values of the coefficient of determination, R^2, are representative. (**d-f**) Residuals of representative fits of bivariate Gaussian approximations to (a-c) by the light-weighting algorithm. (**g-h**) Plots showing dependences of the Gaussian standard deviations (g) w_x and (h) w_y on z position. Uncertainties are comparable to the line width.

Supplementary Table 2. Model functions for localization

<table>
<tr><th>Purpose</th><th>Type</th><th>Fit to</th><th>Form</th><th>Salient output</th></tr>
<tr><td>Lateral localization</td><td>Bivariate Gaussian</td><td>Particle image</td><td>$G_{biv}(x_p, y_p) = A \cdot \exp\left(\frac{-1}{2(1-\rho^2)}\left[\frac{(x_p - x')^2}{w_x^2} - 2\rho\frac{(x_p - x')(y_p - y')}{w_x w_y} + \frac{(y_p - y')^2}{w_y^2}\right]\right) + B$</td><td>$(x', y', \rho, A, w_x, w_y)$</td></tr>
<tr><td>Local lateral localization calibration</td><td>16th-order polynomial</td><td>$x'(z) - x'(z_f)$
$y'(z) - y'(z_f)$
for calibration particles</td><td>$\Delta'_x(z) = \sum_{i=1}^{16} C_i \cdot (z)^i$
$\Delta'_y(z) = \sum_{i=1}^{16} C_i \cdot (z)^i$</td><td>Analytical models for $\Delta'_x(z)$ and $\Delta'_y(z)$ at the location of each calibration particle, $\{\Delta'_x(z)\}_{cal}$ and $\{\Delta'_y(z)\}_{cal}$</td></tr>
<tr><td>Local axial localization calibration</td><td>16th-order polynomial</td><td>$z(\rho_A)$
for calibration particles</td><td>$z(\rho_A) = \sum_{i=1}^{16} C_i \cdot (\rho_A)^i$</td><td>Analytical models for $z(\rho_A)$ at the location of each calibration particle, $\{z(\rho_A)\}_{cal}$</td></tr>
<tr><td rowspan="2">Widefield lateral localization calibration</td><td>Zernike polynomials</td><td rowspan="2">$\{\Delta'_x(z)\}_{cal}$ and $\{\Delta'_y(z)\}_{cal}$</td><td>$\Delta'_x(x', y'; z) = \sum_{i=1}^{400} C_i(z) \cdot Z_i(x', y')$</td><td rowspan="2">Corrections Δ'_x and Δ'_y for lateral position, for the input value of z</td></tr>
<tr><td>Interpolant</td><td>$\Delta'_x(x', y'; z) = \sum_{i=1}^{1100} I_i(x', y') \cdot \{\Delta'_x(z)\}_i$</td></tr>
<tr><td rowspan="2">Widefield axial localization calibration</td><td>Zernike polynomials</td><td rowspan="2">$\{z(\rho_A)\}_{cal}$</td><td>$z(x', y'; \rho_A) = \sum_{i=1}^{400} C_i(\rho_A) \cdot Z_i(x', y')$</td><td rowspan="2">$z$, for the input value of ρ_A</td></tr>
<tr><td>Interpolant</td><td>$z(x', y'; \rho_A) = \sum_{i=1}^{1100} I_i(x', y') \cdot \{z(\rho_A)\}_i$</td></tr>
</table>

x_p: Pixel position in the x direction
y_p: Pixel position in the y direction
A: Gaussian amplitude
ρ: Gaussian correlation coefficient
x': Apparent position of particle in the x direction
y': Apparent position of particle in the y direction
w_x: Gaussian standard deviation in the x direction
w_y: Gaussian standard deviation in the y direction
B: Constant background

z: Particle position in the z direction
cal: Label indicating the set of calibration particles
ρ_A: The shape parameter $\frac{\rho}{A_n}$
C: Polynomial coefficient
Z_i: Zernike polynomial of Noll index i
z_f: z position of best focus for a calibration particle
I: Interpolant weight[1]
Δ'_x: Apparent translation in the x direction
Δ'_y: Apparent translation in the y direction

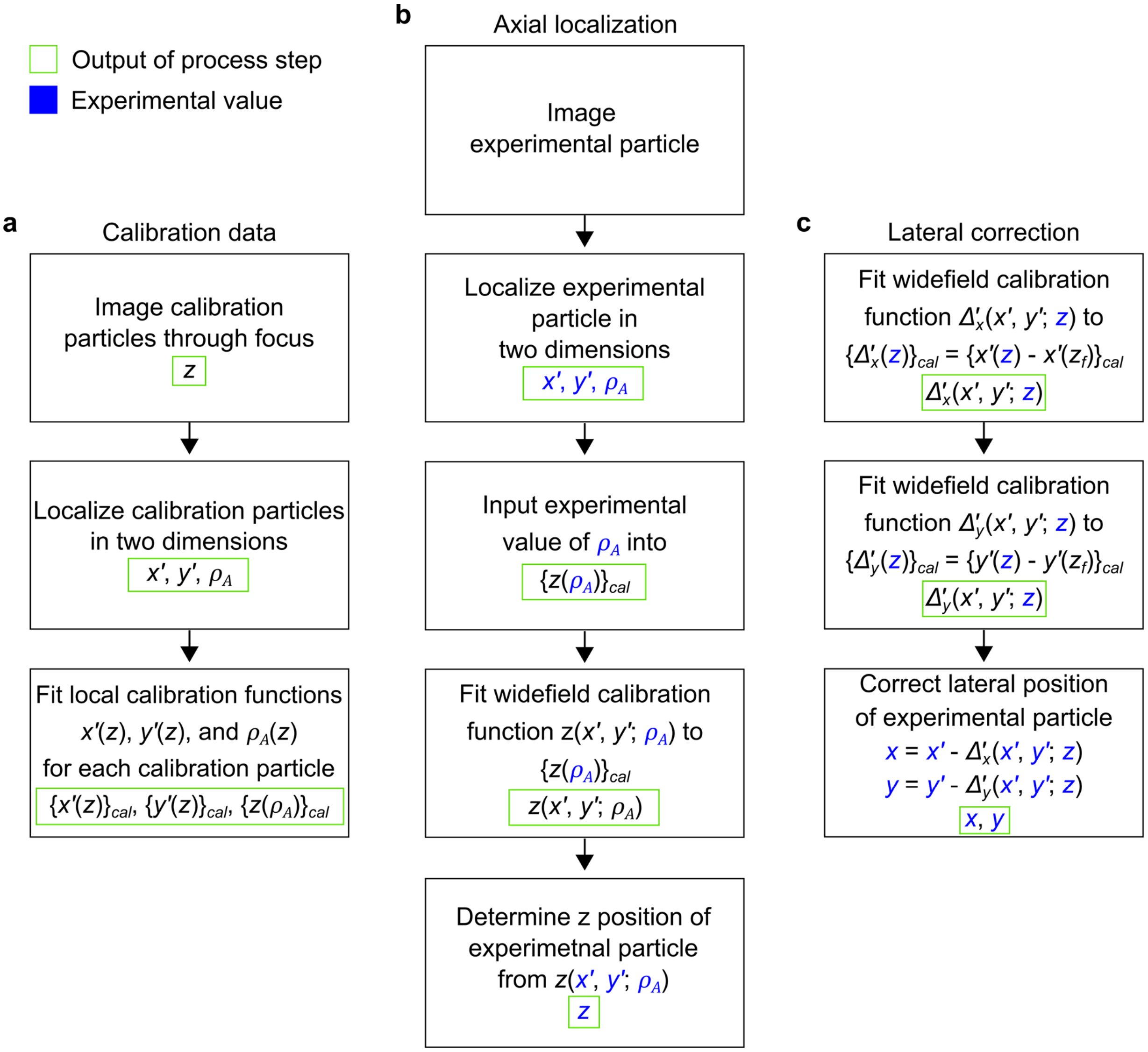

Supplementary Figure 2. Localization scheme. Flow charts showing the process of (**a**) acquiring sets of local calibration functions from calibration particles, (**b**) using local and widefield calibration functions to determine the axial position of an experimental particle, and (**c**) using local and widefield calibration functions to correct the lateral position of an experimental particle.

Supplementary Note 1. Apparent lateral motion

Even for emitters on a planar sample that is normal to the optical axis, field curvature[2] causes apparent variation of axial position, resulting in errors of apparent lateral position. The field curvature for our imaging system is significant (Supplementary Figure 3), although lesser in magnitude than that resulting from an objective lens with a higher value of numerical aperture[2].

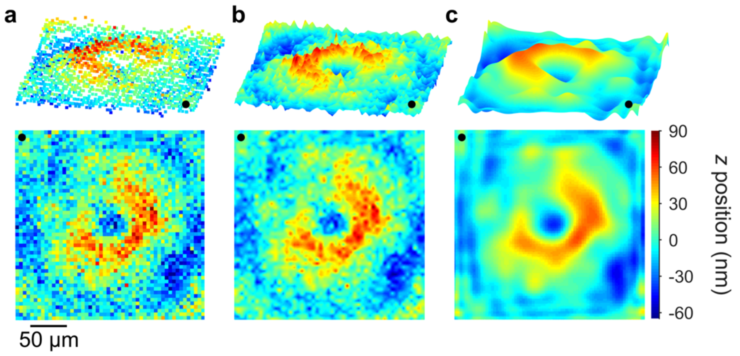

Supplementary Figure 3. Field curvature. Plots showing the surface of best focus from (**a**) the z positions of best focus for each aperture in an array, (**b**) natural-neighbor interpolation between the data in (a), and (**c**), a linear combination of 400 Zernike polynomials fit to the data in (a). We rotate the top plots with respect to the bottom plots for clarity. Black dots indicate the same corners.

If a stationary reference is unavailable, then measurements of apparent lateral motion can include actual lateral motion due to stage drift. An available stationary reference can also exhibit apparent lateral motion with actual axial motion. Both issues can cause errors in the correction of apparent lateral motion. In this study, the calibration data is a combination of 21 data sets from sequential measurements, averaging any systematic effects of stage drift in each measurement. This challenge highlights the benefits of approaches to correct for stage drift that are insensitive to aberration effects, such as direct measurements of stage position. Depending on the requisite accuracy, drift correction by image analysis may be sufficient. Determining suitable reference objects that do not exhibit apparent lateral motion with axial motion is a subject of future work.

Our evaluation of accuracy in correcting apparent lateral motion takes reference values of z position from a piezoelectric actuator, omitting additional error from experimental uncertainty of z position. In an alternate analysis, we treat calibration particles as experimental particles and use the z positions from the combination of local and widefield calibration. The experimental uncertainty in z position (Figure 2f, Figure 5f) propagates through the calibration of apparent lateral motion, but increases the errors of lateral calibration (Figure 5d-e) by less than 0.1 nm for interpolation, and both increases and decreases error, depending on z position, by less than 0.1 nm for Zernike polynomials. An iterative process of determining axial position and correcting lateral position might further improve localization accuracy in all three spatial dimensions.

We could model and correct apparent lateral position as a function of ρ_A instead of z, but doing so provides little benefit and noise in ρ_A complicates the process, such as by requiring sorting of data to establish monotonicity of ρ_A before fitting a local calibration function.

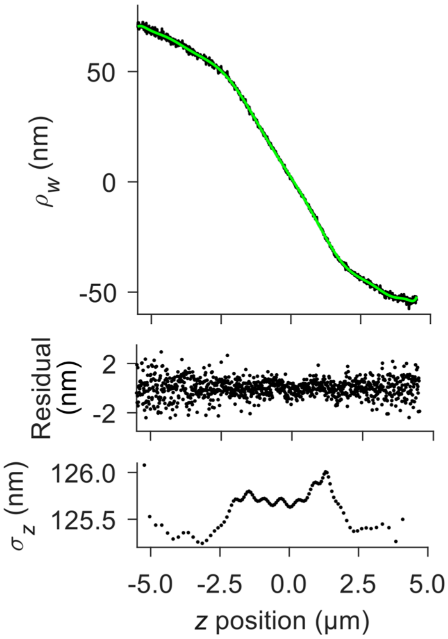

Supplementary Figure 4. Plot showing the dependence of the astigmatic defocus parameter $\rho_w = \rho \cdot \frac{|w_x| + |w_y|}{2}$ on axial position. Fits of bivariate Gaussian models to particle images determine the (black) parameter values and (green line) a polynomial of order 16 models the *z* dependence for axial localization. Residual values in the middle panel indicate uncertainty. Values in the bottom panel are uncertainties of *z* position σ_z from inversion of the polynomial model.

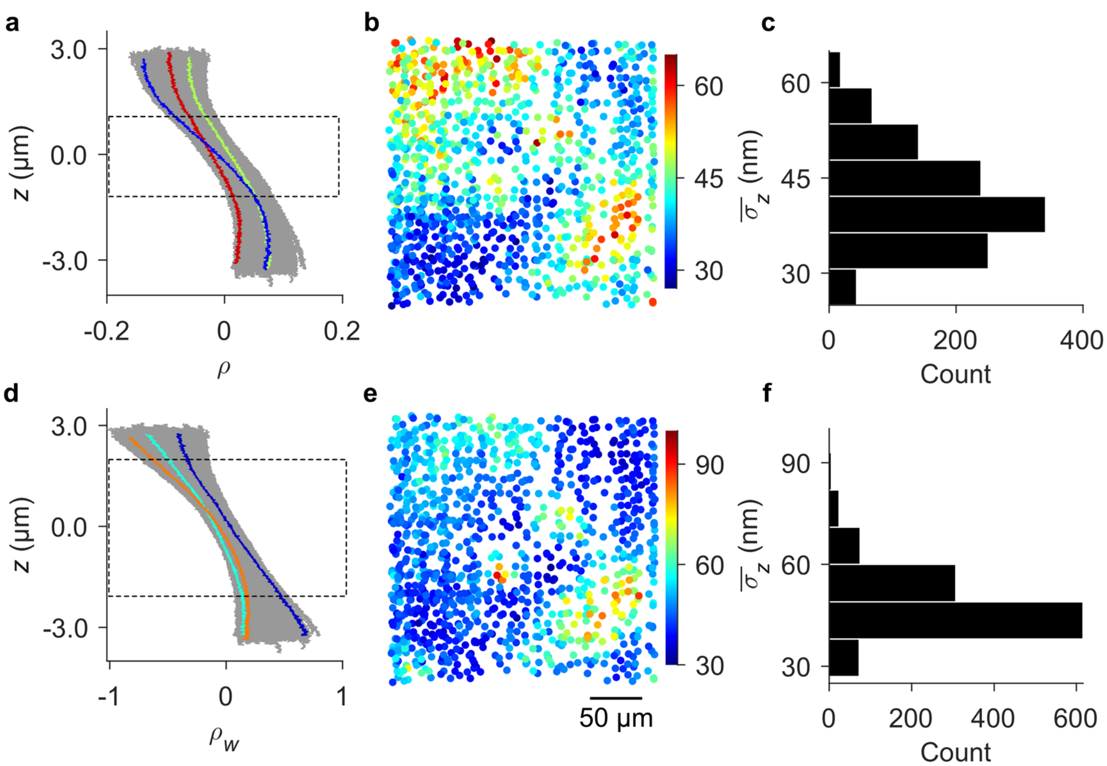

Supplementary Figure 5. Field dependence of local values of uncertainty from axial localization for ρ and ρ_w. (**a, d**) Line plots showing the relationships $z(\rho)$ and $z(\rho_w)$ for many calibration particles. Three representative lines have colors corresponding to the code in (b, e) for local values of uncertainty $\overline{\sigma_z}$ from the polynomial models $\{z(\rho)\}_{cal}$ and $\{z(\rho_w)\}_{cal}$. The overbar denotes the mean value of uncertainty over the axial ranges indicated by the dash boxes, which are (a) 2 µm and (d) 4 µm. (**b, e**) Scatter plots showing the lateral positions of the calibration particles and corresponding values of $\overline{\sigma_z}$ for axial ranges of (b) 2 µm and (e) 4 µm. (**c, f**) Histograms of the data in (b, e) showing asymmetric distributions of $\overline{\sigma_z}$.

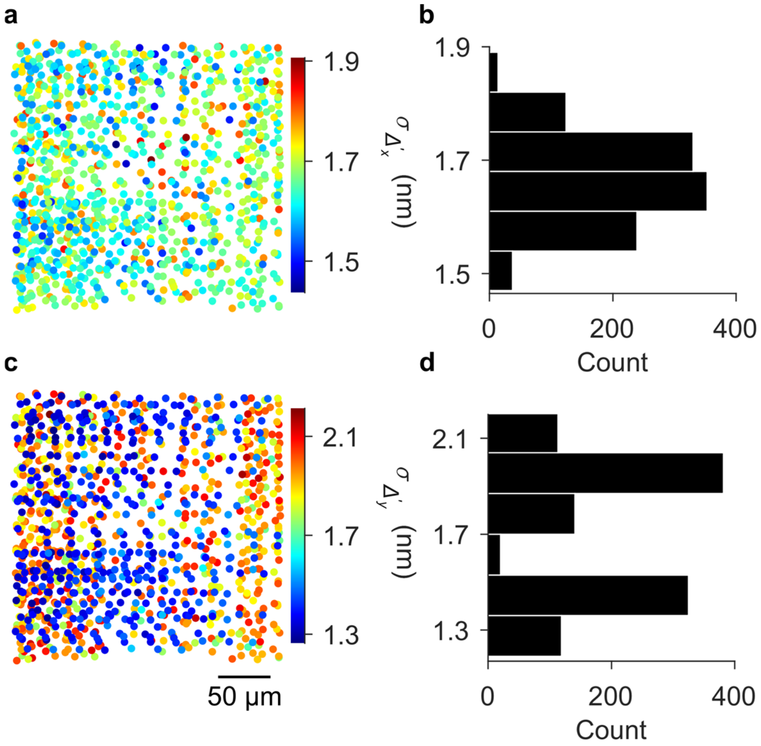

Supplementary Figure 6. Field dependence of local values of uncertainty for Δ'_x and Δ'_y. (**a, c**) Scatter plots showing the lateral positions of the calibration particles and corresponding values of (a) $\sigma_{\Delta'_x}$ and (c) $\sigma_{\Delta'_y}$ for an axial range of 6 μm. (**b, d**) Histograms showing the data in (a, c). The bimodal distribution in (d) indicates a difference between the lateral scan axes of the microscope stage that translates the calibration particles across the lateral imaging field.

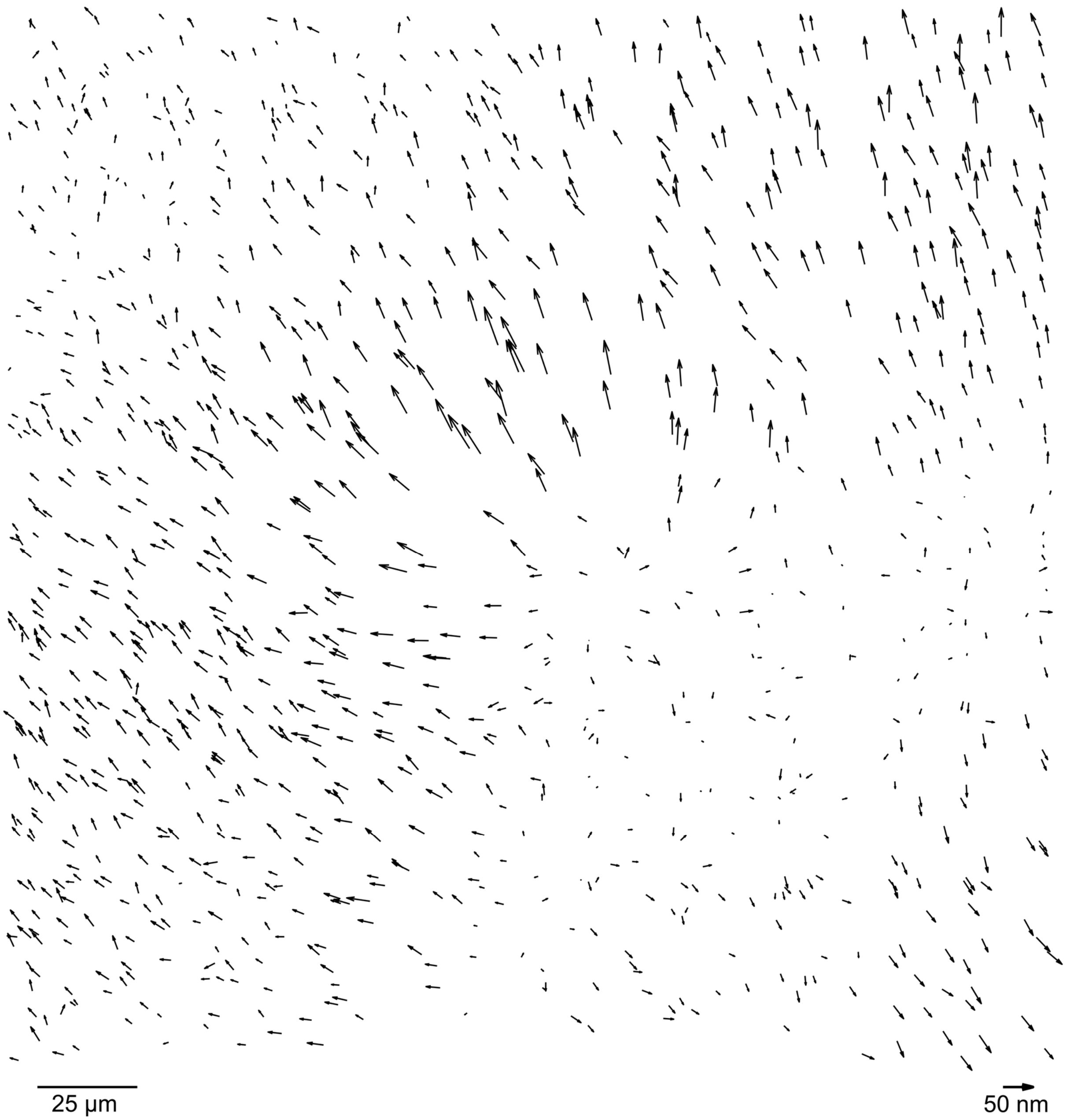

Supplementary Figure 7. Lateral dependence of apparent lateral motion. Vector plot showing the apparent lateral motion of all calibration particles at $z = 2$ μm relative to the focal surface.

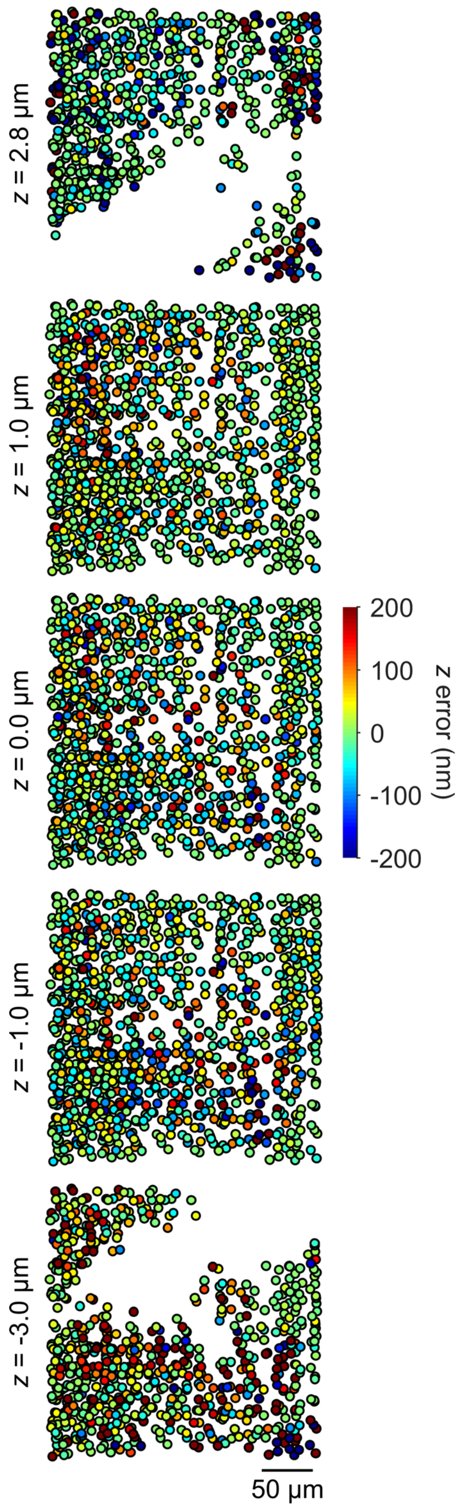

Supplementary Figure 8. Aberrations and sample tilt can limit axial range. Scatter plots showing axial localization errors. Missing data markers indicate calibration particles with values of ρ_A that are outside the range of ρ_A for the other calibration particles. The missing data is due to a combination of the field dependence of ρ_A and the sample tilt, or nutation, of the surface normal of the calibration substrate relative to the optical axis. Lateral position uncertainties are smaller than the data markers. The color scale does not span the full range of values. We can omit such particles from the calibration data, but this limits the polynomial order of the Zernike model or requires a larger axial range for the calibration data to encompass the full experimental range of ρ_A. In contrast, interpolant models require less data to calibrate the same axial range. This is because it is unnecessary to have calibration data spanning the entire lateral extent of the field for every experimental value of the astigmatic defocus parameter.

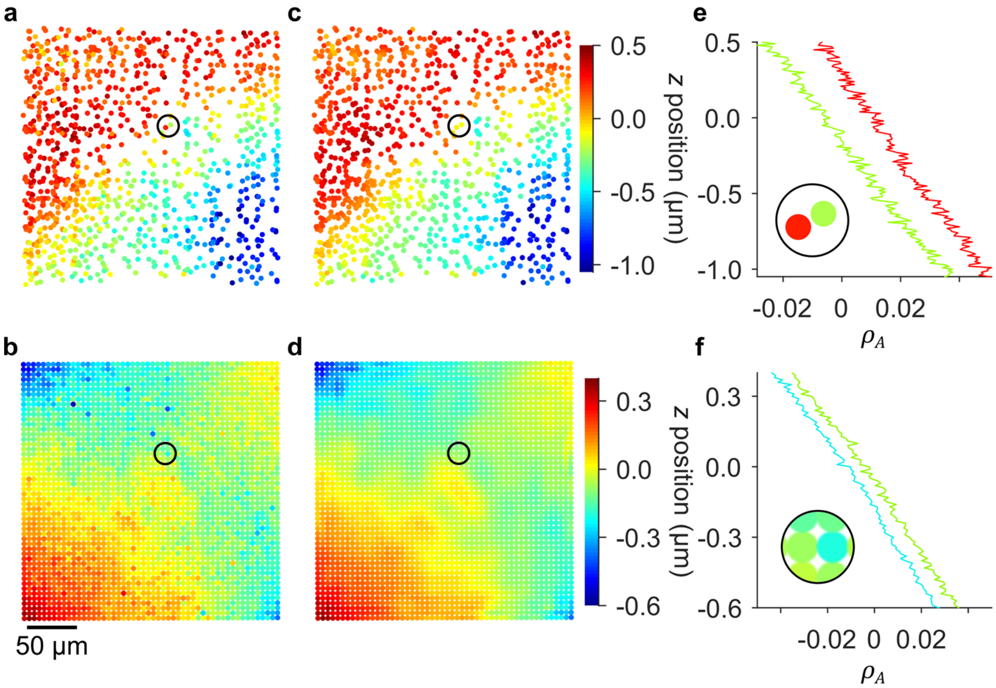

Supplementary Figure 9. Variation of aberration effects at micrometer scales. (**a**, **b**) Scatter plots showing the z positions at which $\rho_A = 0$ for (a) a random array of calibration particles and (b) a square array of subresolution apertures. Black circles are regions of interest. (**c, d**) Scatter plots showing model values of z position from fits of a widefield calibration function of Zernike polynomials to the data in (a, b). Lateral position uncertainties in (a-d) are smaller than the data markers. (**e**, **f**) Plots showing z position as a function of ρ_A for (e) two particles within the region of interest in (a), and (f) two apertures within the region of interest in (b). Insets more clearly show these regions. Variation due to the effects of intrinsic aberrations at this lateral scale of approximately 5 μm produces a significant shift in z. Uncertainties are comparable to those in Figure 2f.

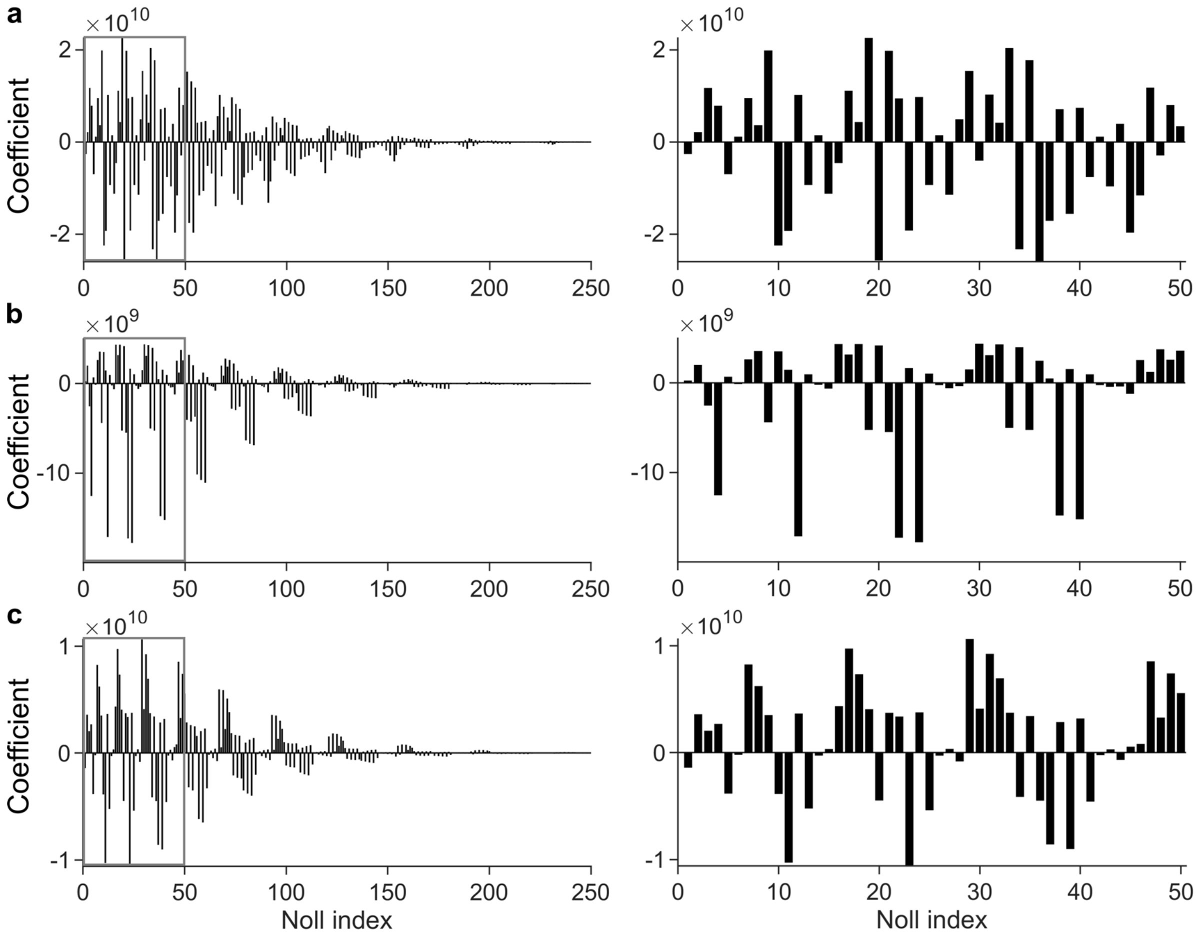

Supplementary Figure 10. Zernike polynomial coefficients. (**a-c**) Plots showing representative coefficients for the first (left) 250 and (right) 50 Zernike polynomials. The gray boxes in the left plots bound the first 50 coefficients in the right plots. The coefficients are from widefield calibration functions for (a) $\rho_A = 0.1$, (b) $\rho_A = 0.0$, and (c) $\rho_A = -\ 0.1$, and are in order of Noll index.

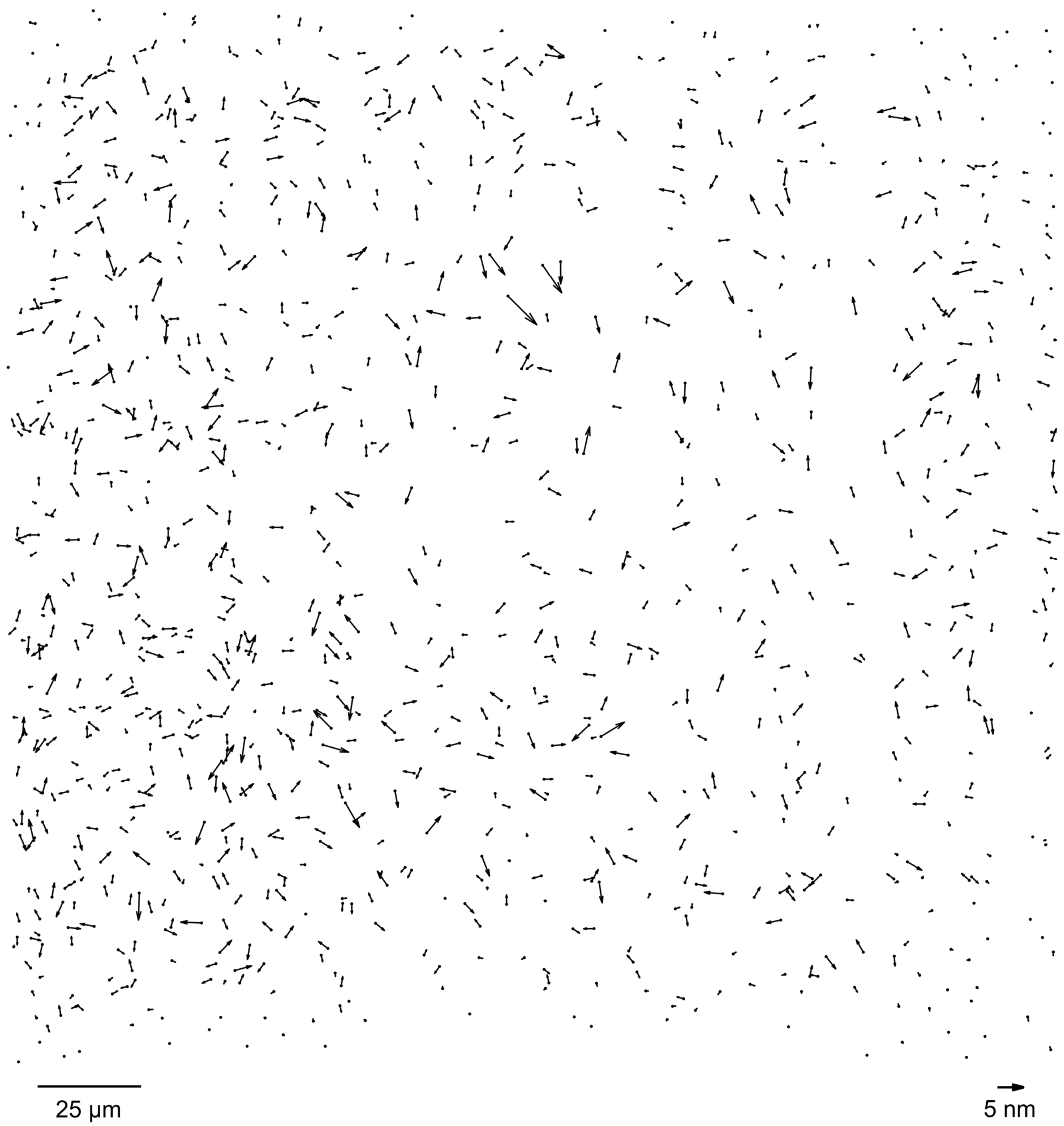

Supplementary Figure 11. Lateral widefield calibration errors for Zernike polynomials. Vector plot corresponding to the data in Figure 5a-b.

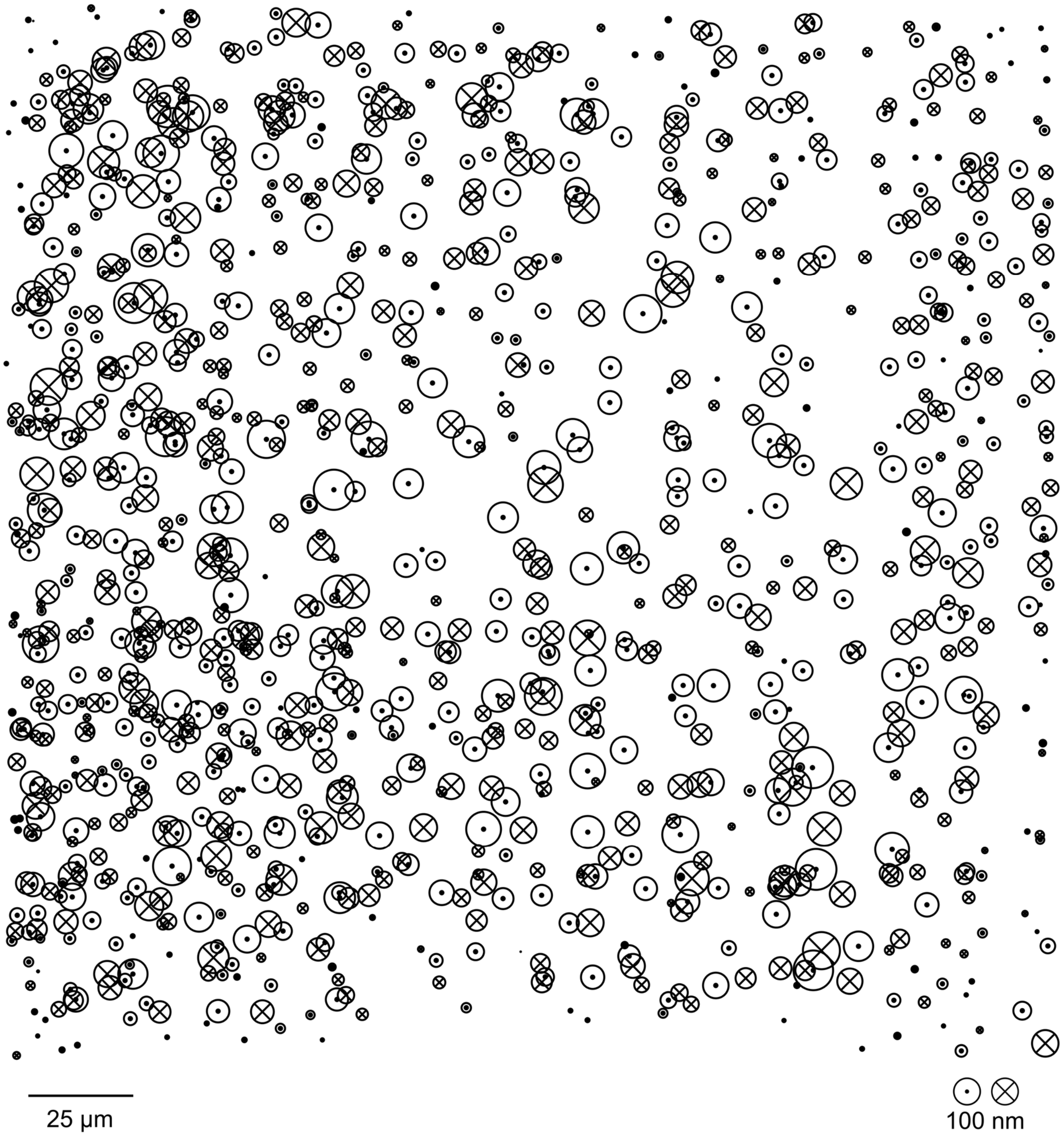

Supplementary Figure 12. Axial widefield calibration errors for Zernike polynomials. Vector plot corresponding to the data in Figure 5c. A circle with a dot points toward the viewer and a circle with a diagonal cross points away from the viewer.

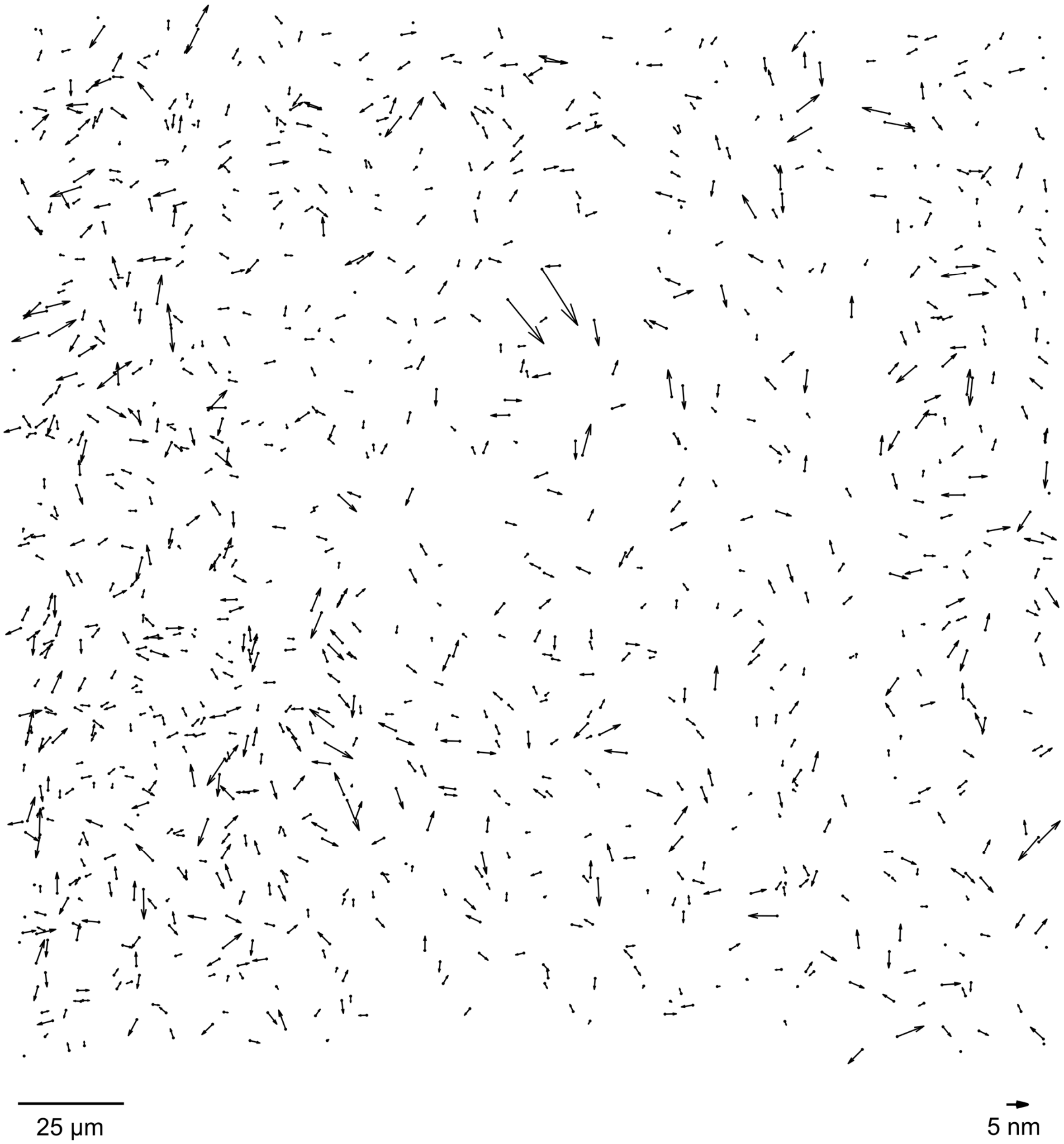

Supplementary Figure 13. Lateral widefield calibration errors for natural neighbor interpolation. Vector plot showing errors for a representative value of $z = 0$ relative to the focal surface.

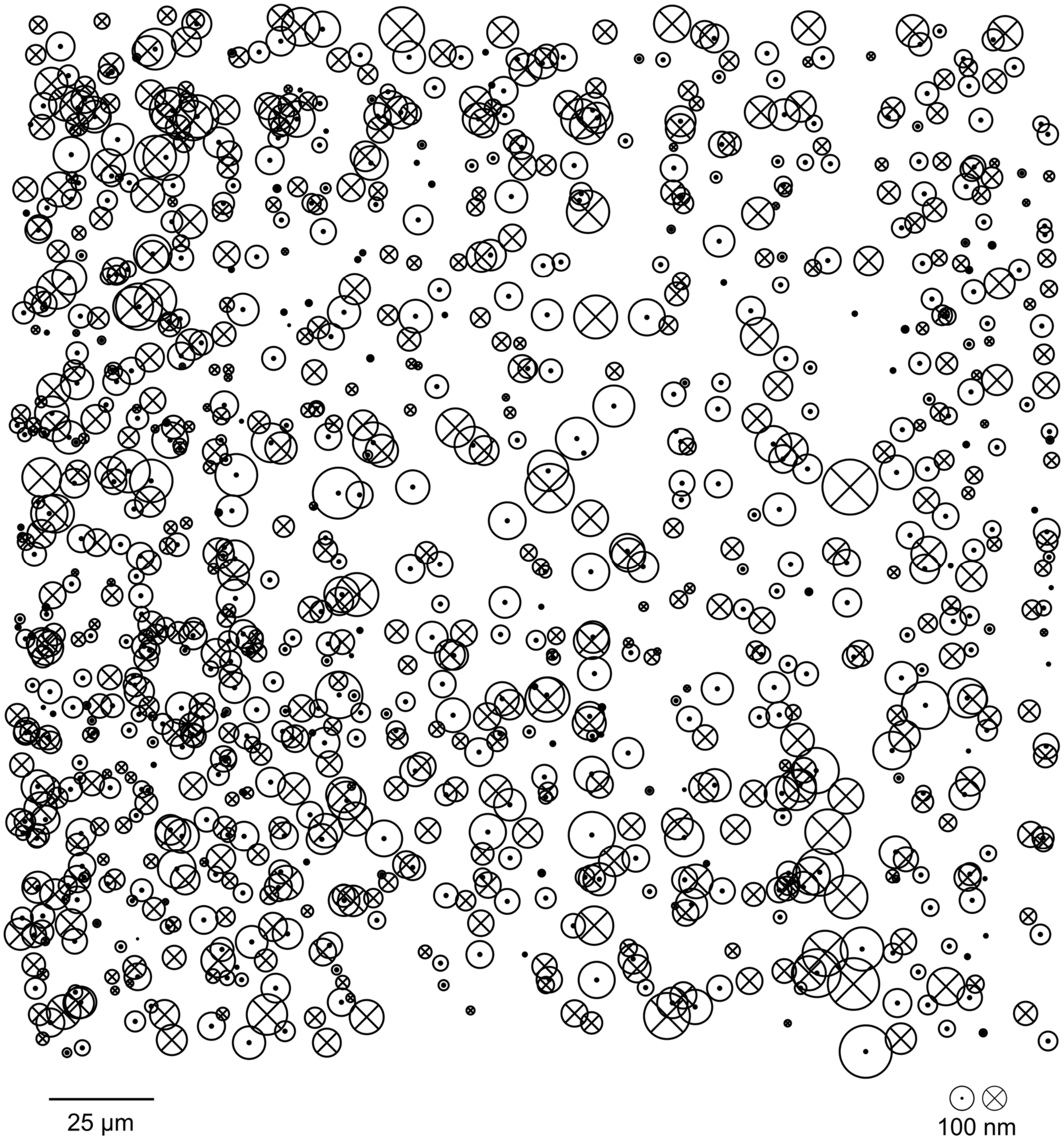

Supplementary Figure 14. Axial widefield calibration errors for natural neighbor interpolation. Vector plot showing errors for a representative value of $z = 0$. A circle with a dot points toward the viewer and a circle with a diagonal cross points away from the viewer.

Supplementary Note 2. Rigid transformation model

Accuracy of rigid transformations and particle tracking

A comparison of the residuals of the rigid transformation to the errors that we expect from the uncertainty of localizing single particles confirms the accuracy of the rigid transformation and our method of particle localization and tracking. In the x and y directions, the residuals of the rigid transformation are approximately equal to the sum of the random error from shot noise, pixelation, and background noise (Supplementary Figure 15a-b, gray data), as well as the systematic error from apparent lateral motion (Supplementary Figure 16, red data), which includes any fitting errors from model mismatch. Even with slight deviations from rigidity in the z direction (Supplementary Figure 15c-e), the residuals are approximately equal to the sum in quadrature of the two main uncertainties in our method of axial localization, from local and widefield calibration. The former uncertainty includes the effects of shot noise, pixelation, and background noise. This agreement indicates the absence of additional errors that any photobleaching of the fluorescent particles would cause in axial localization by the parameter ρ_A. Errors from apparent lateral motion and widefield calibration of axial localization vary over the tracking range in z. Therefore, for this comparison (Supplementary Table 3), we consider mean values of root-mean-square error in the range of -1.5 μm $< z <$ 2 μm, bounding the range of the experimental particles.

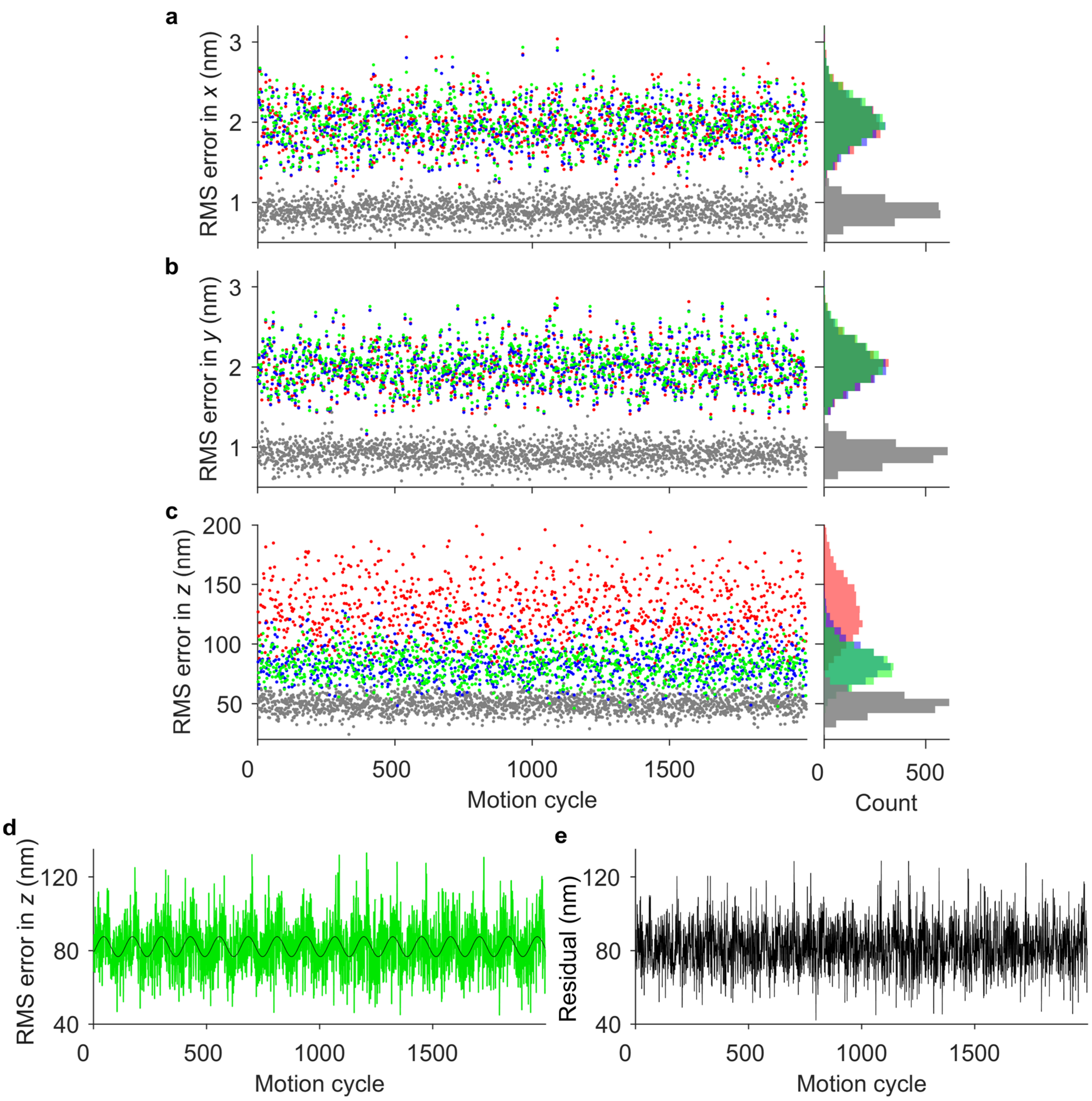

Supplementary Figure 15. Residuals of rigid transformations and uncertainties of single particles. Scatter plots and histograms showing root-mean-square residuals of rigid transformations from calibration by (green) Zernike polynomials, (blue) natural neighbor interpolation, and (red) nearest neighbor interpolation in the (**a**) *x*, (**b**) *y*, and (**c**) *z* directions. The plots show data at half density. (gray) Empirical localization precision for nominally static single particles on the load gear, without actuation of microsystem. Combining the information from the 28 particles on the gear in a rigid transformation reduces uncertainties to the values in Table 1. (**d**) Line plot showing the green data in (c) to clarify the periodic fluctuation. These data have an excess kurtosis of 0.19 ± 0.11. Fitting these data to a (black) sine function yields an amplitude of 5.4 nm ± 0.4 nm and a period of 127.9 motion cycles ± 0.3 motion cycles. (**e**) Plot showing the residuals of the fit in (d), with an excess kurtosis of 0.14 ± 0.11, showing that a simple model and analytical removal of the periodic fluctuation results in residuals of the rigid transformation that are slightly closer to normal.

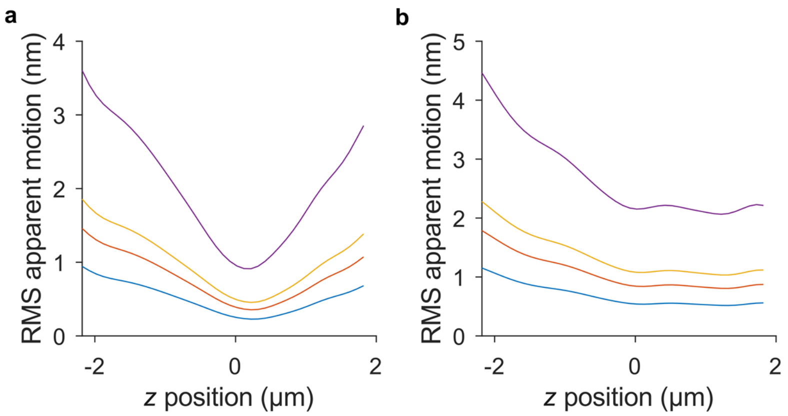

Supplementary Figure 16. Apparent lateral motion. (**a-b**) Plots showing the root-mean-square apparent motion of single particles in the (a) x and (b) y direction produced by a change in z position of (purple) 200 nm, (orange) 100 nm, (red) 78 nm, and (blue) 50 nm, as a function of z position.

Supplementary Table 3. Comparison of transformation residuals and localization uncertainty

	Shot noise (nm)	Apparent motion (nm)	Total (nm)	Residuals (nm)
x	0.9	1.0[a]	1.9	2.0
y	0.9	1.0[a]	1.9	2.1

	Local calibration (nm)	Widefield calibration (nm)	Total (nm)	Residuals (nm)
z	50	71[b]	87	83

[a]These values are approximate mean values from the experimental range -1.5 μm < z < 2.0 μm of Supplementary Figure 16
[b]This value is the mean from the experimental range -1.5 μm < z < 2.0 μm in Figure 5f

Uncertainty of motion measurements in six degrees of freedom

We evaluate the uncertainty of our motion measurements using Monte-Carlo simulations, propagating the total experimental localization uncertainties of single particles through the rigid transformation in three dimensions. This evaluation of uncertainty provides values that are specific to the particular motion in an experiment with a particular coordinate system and constellation of particles. We simulate the gear motion by applying the experimental transformations in series to the particle positions in the first micrograph of the measurement series, producing a series of particle positions that are identical to the experimental data in all aspects except for the effects of noise due to localization uncertainty. In the experimental data, this noise produces residual errors in the one-to-one mapping of particle positions from the rigid transformation (Supplementary Figure 15). We add comparable noise to the synthetic particle positions, drawing random values from normal distributions with means and variances corresponding to the means and variances of the experimental residuals for each particle. Additional variance of 8 % to 15 % is necessary to match the experimental residuals, possibly due in part to the systematic deviations from normality in the experimental data (Supplementary Figure 15). Representative data from one simulation are in Supplementary Figure 17 and Supplementary Table 4. We measure the synthetic motion in the presence of this additional noise by fitting rigid transformations, and we define measurement errors as the difference between this motion and the true motion in the absence of noise. Representative distributions of these errors are in Supplementary Figure 17. Finally, we pool the standard deviations of these measurement errors from 10,000 simulations (Table 1) to obtain an uncertainty component which corresponds to a 68 % coverage interval.

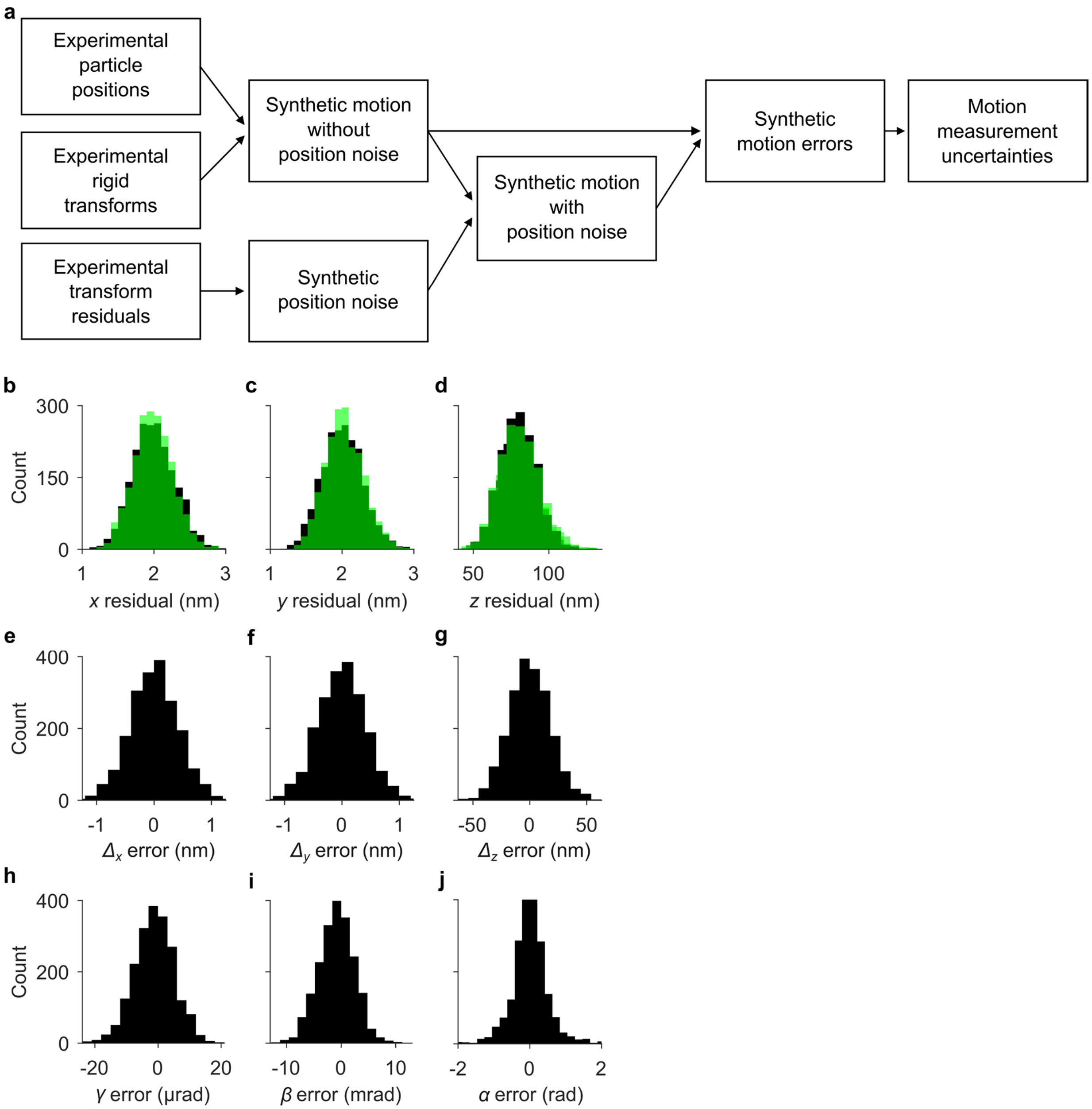

Supplementary Figure 17. Uncertainty evaluation for rigid transformations. (**a**) Flowchart showing our process of evaluating uncertainty in motion measurements by rigid transformations. The purpose of this process is to reproduce the experimental data in a simulation in which we know the motion. (**b-d**) Histograms showing residuals in the (b) x, (c) y, and (d) z directions from fitting rigid transformations to (green) experimental and (black) synthetic positions of particles. Root-mean-square values are in Supplementary Table 3. The evident agreement indicates that the simulation is representative of the experiment. (**e-i**) Histograms showing measurement errors for (e) Δ_x, (f) Δ_y, (g) Δ_z, (h) γ, (i) β, and (j) α from a representative simulation. We pool the standard deviations of these measurement errors from 10,000 simulations to obtain an uncertainty component which corresponds to a 68 % coverage interval.

Any unintentional motion of the microscope system, which occurs in a common mode for all experimental particles, can produce translation relative to the imaging sensor that is consistent with rigid translation of the gear. However, such motion is not a result of the intentional operation of the microsystem, constituting another potential component of error for translations but not for rotations[4]. We estimate the error resulting from unintentional motion of the microscope system, by the apparent translations of the centroid of the particle constellation in the absence of intentional motion of the load gear. These components sum in quadrature, giving the total values in Table 1.

Supplementary Table 4. Transformation residuals from a representative simulation

Degree of freedom	Experimental RMS residuals (nm)	Synthetic RMS residuals (nm)
x	2.04	2.02
y	2.09	2.03
z	83.3	80.8

Supplementary Note 3. Field corrections

Distortion

In a previous study, we showed that localization measurements in the two lateral dimensions can manifest large errors due to nonuniform magnification across the lateral field, and that such distortion can vary with axial position[2]. In the present study, our objective lens has a relatively low value of numerical aperture of 0.55. We find that this lens has relatively low distortion, resulting in errors in the rigid transformation that are consistent with the errors that we expect from photon shot noise in combination with localization errors and the field dependence thereof (Supplementary Figure 15). Therefore, calibration of the mean value of image pixel size, which still deviates substantially from the nominal value, is sufficient in the present study.

Apparent lateral motion in microsystem tracking

Counterintuitively, the rigid transformation model achieves better accuracy without correcting for the apparent lateral motion that results from axial motion of each particle. This is because single motion cycles produce relatively small displacements in z of each particle, with a mean value of 78 nm (Supplementary Figure 16). For such displacements, the contribution of the apparent but erroneous lateral motion to the residuals of the rigid transformation (Supplementary Figure 15) is less than the uncertainty of correcting the apparent lateral motion (Figure 5d-e). Therefore, we omit this correction of lateral position for our specific application of microsystem tracking by a rigid transformation, although this correction remains generally necessary.

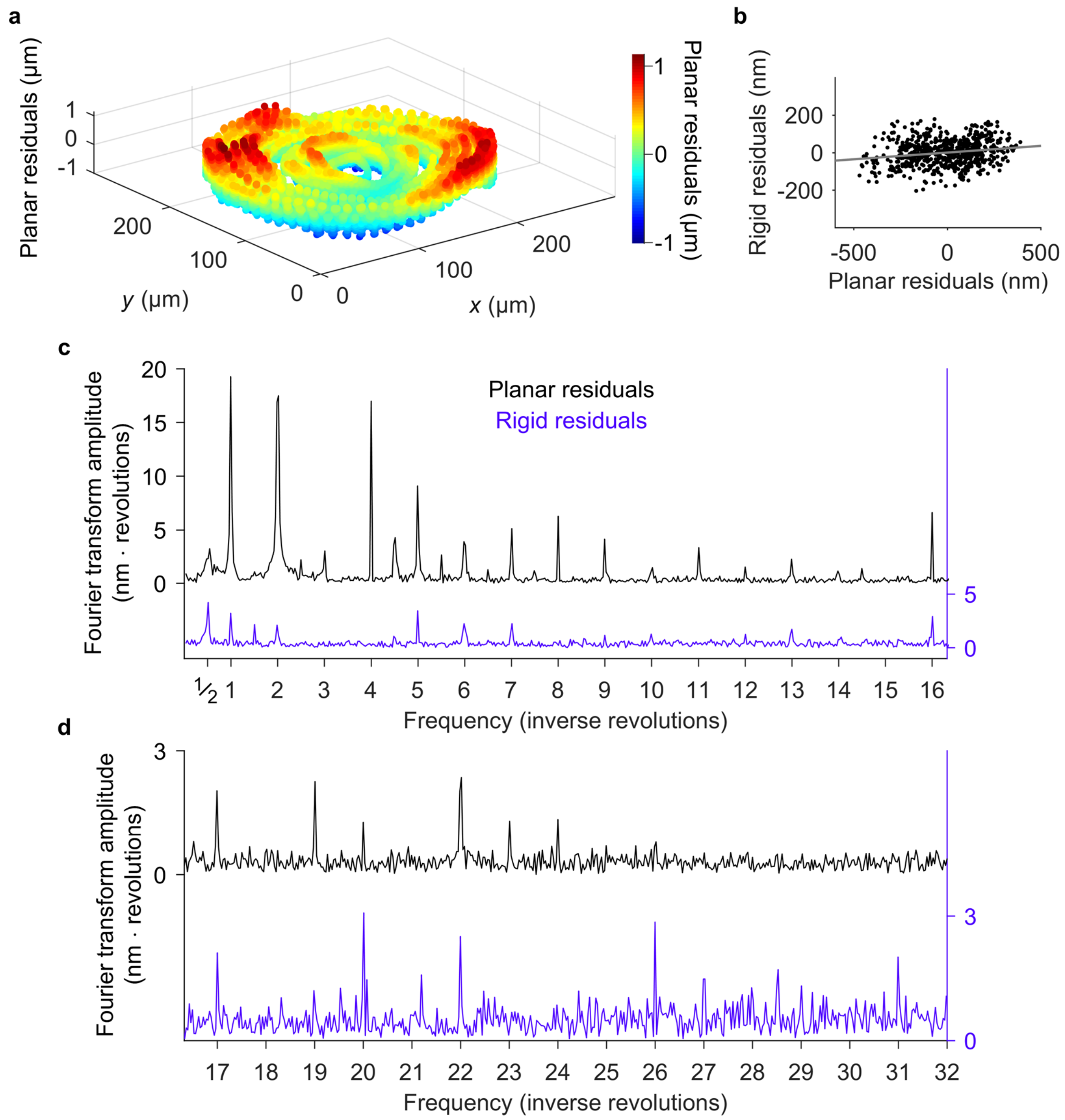

Supplementary Figure 18. Flexure of the load gear. (**a**) Scatter plot showing the residuals of fitting a plane to all particle positions over all 2000 motion cycles. (**b**) Scatter plot showing rigid residuals as a function of planar residuals for a representative particle with (gray line) a linear fit showing a positive correlation. We reduce the data density by a factor of three for clarity. (**c-d**) Plots showing Fourier transformations of the root-mean-square residuals in the z direction from fitting (black) planes and (violet) rigid transformations to the particle positions following each motion cycle. For clarity, we split the range of frequencies between (c) and (d). Prominent peaks in (c-d) correspond to 128, 64, 32… motion cycles, with 64 motion cycles driving the gear through one complete revolution.

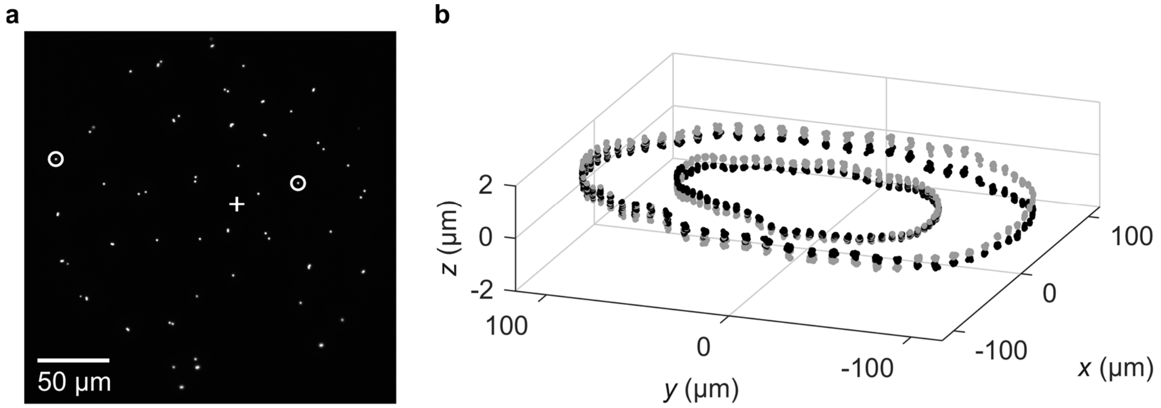

Supplementary Figure 19. Rotational play produces a reciprocating vertical shift. (**a**) Fluorescence micrograph at experimental magnification showing a constellation of fluorescent particles on the surface of the load gear. The cross indicates the centroid of the subset of particles that we use for tracking, and the circles indicate two representative particles on opposing sides of the gear hub. (**b**) Scatter plot showing the motion of the circled particles in (a). Gray and black data markers indicate odd and even numbers of gear revolutions, respectively. Uncertainties are smaller than the data markers.

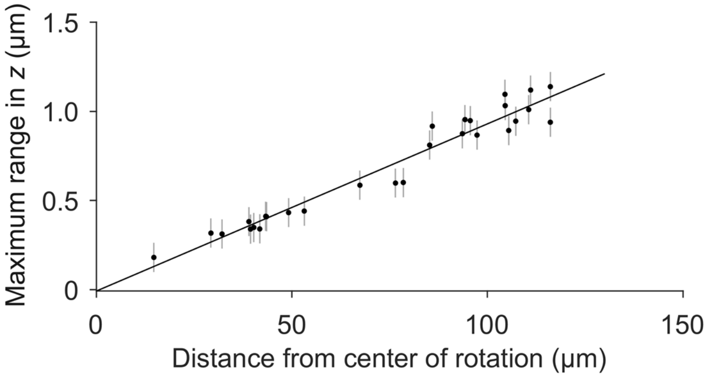

Supplementary Figure 20. Rotational play out of the plane. Plot showing the maximum range in z position over all nominal locations for each particle, as a function of the particle distance from the center of rotation in the nearly parallel planes of the microsystem substrate and imaging sensor. The slope of a linear fit gives the rotational play out of the plane as 9.38 mrad ± 0.44 mrad. Uncertainties are the root-mean-square of the root-mean-square errors in Supplementary Figure 15c.

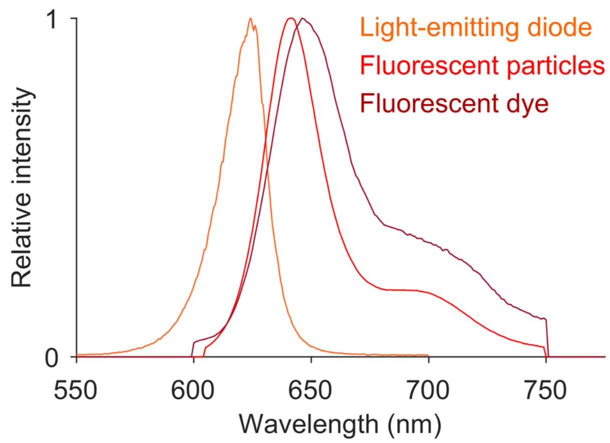

Supplementary Figure 21. Emission spectra. Plot showing representative spectra of the (orange) light-emitting diode, (red) fluorescent molecules, and (dark red) fluorescent particles. The data for the fluorescent molecules and particles are from the manufacturer.

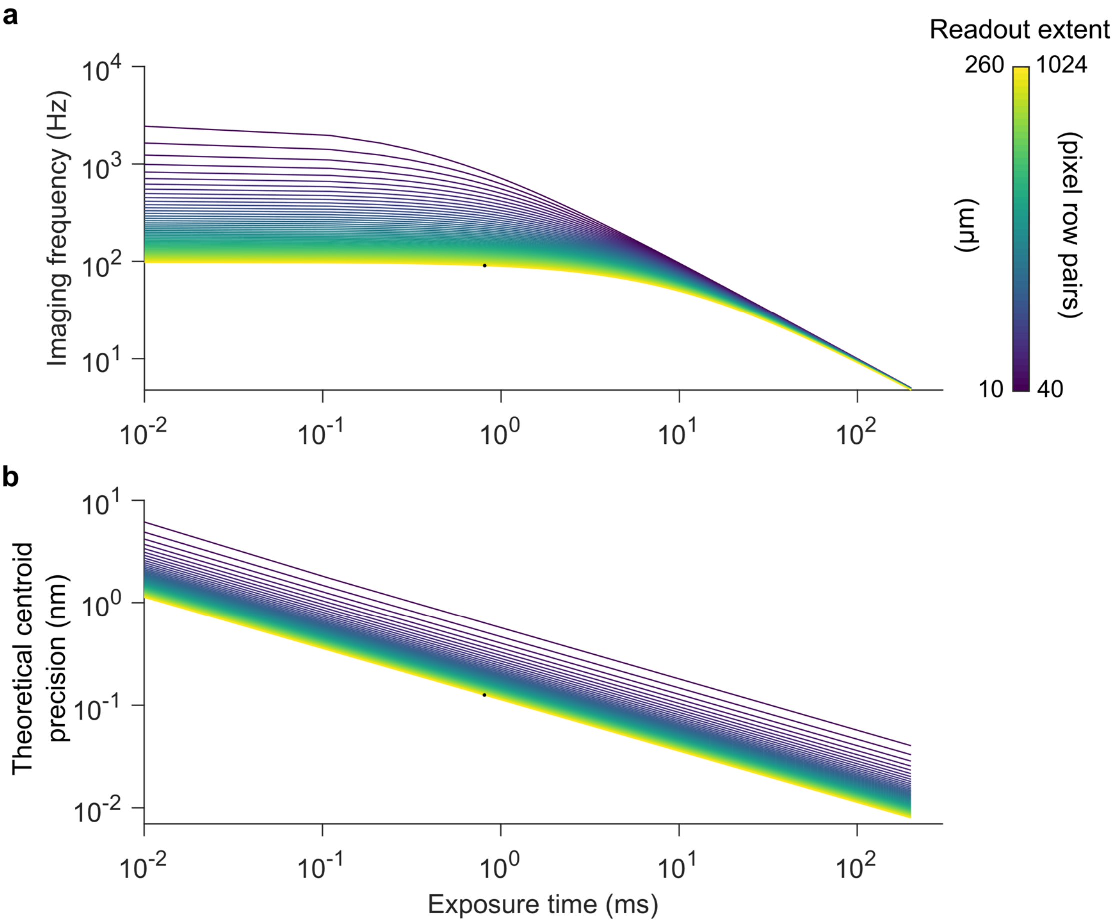

Supplementary Figure 22. Effect of readout extent on imaging frequency and centroid precision. (**a-b**) Plots showing (a) imaging frequency and (b) theoretical centroid precision[3] as a function of exposure time, for different readout extents of a CMOS imaging sensor operating with a global shutter. Black dots indicate the experimental parameters in this study. The calculation in (b) involves the simplifying approximation of a uniform number of signal photons per unit area per unit time across the lateral imaging field. Across the maximum area of the field, the number of signal photons per unit time is equivalent to that of a constellation of 28 particles in our micrographs. Reducing the field size by reducing the readout extent increases the imaging frequency but decreases the total number of signal photons. This approximates how a smaller field limits the number of emitters that can contribute signal photons to a rigid transformation.

Supplementary References

1. Sibson, R. A brief description of natural neighbor interpolation. *Interpreting multivariate data*, 21-36 (1981).
2. Copeland, C. R. *et al.* Subnanometer localization accuracy in widefield optical microscopy. *Light: Science & Applications* **7** (2018).
3. McGray, C., Copeland, C. R., Stavis, S. M. & Geist, J. Centroid precision and orientation precision of planar localization microscopy. *Journal of Microscopy*, 238-249 (2016).
4. Copeland, C. R., McGray, C. D., Geist, J., Aksyuk, V. A. & Stavis, S. M. Transfer of motion through a microelectromechanical linkage at nanometer and microradian scales. *Microsystems & Nanoengineering* **2** (2016).
