# Supplementary material for: Accurate localization microscopy by intrinsic aberration calibration
Source: Nat Commun. 2021 Jun 24;12:3925. doi: 10.1038/s41467-021-23419-y (PMC8225824; doi:10.1038/s41467-021-23419-y)
Supplement: Supplementary file 3 — Description of Additional Supplementary Files [file 41467_2021_23419_MOESM3_ESM.pdf]

Description of Additional Supplementary Files

Supplementary Movie 1: Play from clearance between gear teeth. Brightfield micrographs showing variation in relative positions of meshing gear teeth during actuation.

Supplementary Movie 2: Play from clearance between load gear and hub. Brightfield micrographs showing translation of the load gear about the hub during actuation.

Supplementary Movie 3: Animation of load gear motion. The animation magnifies motion in the z direction by a factor of 10 for emphasis.
